# Superiority of biomimetic micelle-entrapped nanoporous silica xerogel to deliver poorly water-soluble itraconazole

**DOI:** 10.1039/d2ra04698a

**Published:** 2022-10-07

**Authors:** Xuejun Li, Dongyan Li, Zhining Liu

**Affiliations:** Ultrasound, The First Affiliated Hospital of Jinzhou Medical University Jinzhou China ningningjin0607@163.com; Department of Hematology, The First Affiliated Hospital of Jinzhou Medical University Jinzhou China; Department of Human Source, The First Affiliated Hospital of Jinzhou Medical University Jinzhou China

## Abstract

Micelle-entrapped silica xerogel (M-Silica xerogel) was biomimetically synthesized to combine the advantages of micelles and silica xerogel to load poorly water-soluble drug itraconazole (ITZ). Tween 20, tween 40, and tween 80 were applied to prepare micelles as the templates for M20-Silica xerogel, M40-Silica xerogel, and M80-Silica xerogel, respectively. During the silica frame construction, the surfactant formed a micelle as the porous template, silicon hydroxyl groups interacted with the hydrophilic parts of the micelle, and polyethylenimine catalyzed silica polycondensation owing to its amino groups, resulting in the formation of the M-Silica xerogels. The results showed that the particle size of the sub-particles from the M40-Silica xerogel was larger than from the M20-Silica xerogel, and the M80-Silica xerogel was the largest among these three samples, demonstrating that the emulsifying ability had a direct impact on the particle size of the M-Silica xerogel. The M-Silica xerogel had a large pore size in the range of 10–50 nm. Small mesopores (2–5 nm) dominated the pore size of the M20-Silica xerogel, while medium mesopores (5–10 nm) occupied most the pore distribution of the M40-Silica xerogel, and large mesopores (10–50 nm) shouldered most the pore distribution for the M80-Silica xerogel. Among these three drug-loaded carriers, the M40-Silica xerogel with the largest amount of medium mesopores presented the best ITZ-release behavior, demonstrating that medium mesopores facilitated drug release, while small mesopores impeded drug release and large mesopores were not favorable to retaining amorphous drugs in the pores.

## Abstract 2

Micelle-entrapped silica xerogel (M-Silica xerogel) was biomimetically synthesized to combine the advantages of micelles and silica xerogel to load poorly water-soluble drug itraconazole (ITZ).

## Graphical abstract

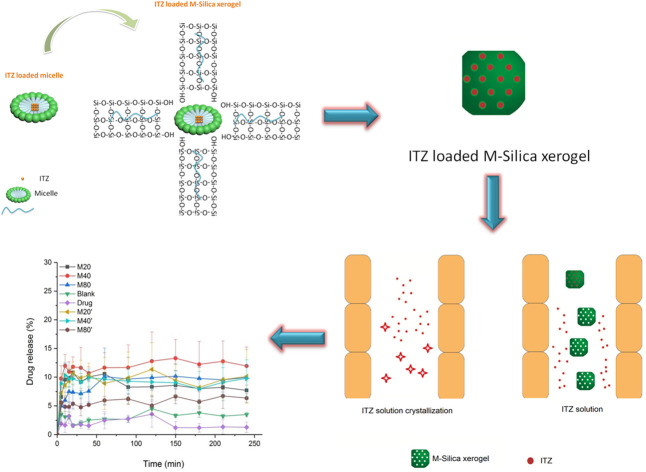

## 1. Introduction

In recent years, the medical research area has experienced tremendous development based on the rapid growth of modern technology and enhancement in people's demand. Many drug candidates with good pharmacological activity have been discovered, but a considerable part of them are limited by their low solubility,^1^ making these drug candidates less absorbed by the human body and also bringing a lot of difficulties in clinical applications. Therefore, the solubility of water-insoluble drugs has attracted the attention of scientific researchers.^2^ Many strategies have been developed to increase their solubility, such as solid dispersions,^3^ a cyclodextrin complex, phospholipid complex, and self-emulsification.^4^ However, many serious problems occur in the working process, which significantly limit their application. The appearance of silica carrier opens up a new way to solve the problem of the low solubility of water-insoluble drugs.^5^ A silica carrier with a large specific surface area, non-toxicity, good biocompatibility, flexible surface modification, low cost of preparation, and high drug-loading efficiency can be used as a kind of ideal drug carrier. The earliest explored silica carrier was MCM-41 with ordered mesopores that was synthesized in 1992,^6^ followed by the SBA series and HOM series. Drug molecules form hydrogen bonding with the silanol group, which can improve drug powder dispersion and wettability. The loaded drug phase changes from crystalline to amorphous to obtain a higher free energy and greater mobility.^7–9^ Initially, the silica carrier was applied for controlled-release formulations and later for water-insoluble drugs with an aim to improve water solubility. Drug-loading methods, drug-loading capacity, as well as *in vitro* and *in vivo* drug-delivery effects have all been widely studied. In the current stage, the solubility of many drugs, including paclitaxel, cirotazole, and rographolide, have been improved with the help of utilizing a silica carrier.^10–14^

Because of their large specific surface area, silica carrier materials can serve as an excellent reservoir for storing hydrophobic drugs, and the silica carrier material can be degraded into simple silyl units *in vivo* and removed from the main body.^15–17^ For water-insoluble drugs, the drug is usually adsorbed into the carrier with an amorphous or molecular form. In addition, silica carrier materials modified by different functional groups can not only change their hydrophobicity, but also their adsorption capacity and cellular affinity.^18^ Zhang^19^ used a new silica carrier to encapsulate the water-insoluble drug telmisartan. The results showed that under the optimal conditions, the drug-loading capacity could reach up to 60%, and the dissolution rate of the drug was significantly improved. Hollow mesoporous silica was synthesized using hard-template phenolic-resin nanoparticles. The study showed that the hollow mesoporous silica could perform as a high drug-loading carrier for regulating insoluble drug release, which could achieve sustained release.^20^ The widely studied application of silica carriers can provide great help for solving the problems of the low solubility and poor bioavailability of water-insoluble drugs.

As a carrier of nanomaterials, micelles^21^ have attracted great attention because of their various outstanding advantages. Polymer micelles have a wide range of drug-loading capabilities, good tissue permeability, and a long retention time in the body, which can effectively help drugs reach the target. Moreover, polymer micelles have the characteristics of nanomaterials because of their small particle size. Further, the micelle solution is small in size, and it cannot be easily eliminated by the liver, excreted by the kidneys, or easily absorbed by reticuloendothelial cells, thus prolonging the circulation time of drugs in the blood and making them good potential pharmaceutical nanoccarriers.^22^ Polymer micelles are thus widely used in pharmacy, whereby they can be used as carriers of antitumor drugs, anti-inflammatory drugs, antifungal drugs, antisedatives and hypnotic drugs, and antipsychotic drugs.^23–25^ For antitumor drugs, adriamycin polymer micelles were prepared and administered to leukemia rats for pharmacological tests. The results showed that the toxic dose of adriamycin was 30 mg kg^−1^, while the toxic dose of micelles reached up to 600 mg kg^−1^, and the survival time of the rats was significantly prolonged.^26^ Therefore, it is obvious that micelles can be rendered as an excellent nanomaterial due to their superiority in delivering drugs.^27^

In recent years, silica with a nanoporous structure has been obtained by applying biomimetic templates, including polyamine,^28^ proteins, and enzymes, and this synthesis method has the advantages of mild working conditions and easy controllability of the silica structure. The present study facilely combined the advantages of nanoporous silica synthesized using a biomimetic method and micelles to establish a novel nanoporous silica drug-delivery system, namely a micelle-entrapped nanoporous silica xerogel (M-Silica xerogel). The related reports for this type of silica xerogel are rare in the current stage. The poorly water-soluble drug itraconazole (ITZ) was chosen as the model drug. ITZ belongs to a classical antifungal drug family and it is applied in the gynecology department to treat candida infection. It is widely known that a lot of women suffer from candida infection within a one-year period and ITZ is commonly used in clinical treatment. However, the poor water-soluble property of ITZ lowers its oral absorption and reduces its treatment effect. As is also known, ITZ can have negative inotropic effects and can even lead to heart failure with a reduced ejection fraction. Heart failure is more frequently seen in those receiving a total daily dose of 400 mg. Therefore, there is an urgent need to improve the drug release and absorption with an aim to lower the drug's side effect for causing heart failure.^29–31^ To solve this problem, the M-Silica xerogel was used as an ITZ carrier and the loaded water-insoluble drug was found to be amorphous in the M-Silica xerogel, and the entrapped micelles exerted certain functions to avoid drug re-crystallization in the system. Furthermore, the characteristics of the M-Silica xerogel as well as its drug-release management principles were systemically investigated, which could give a strong foundation for the further study of silica xerogel drug-delivery systems. A complementary aspect of this study is the breakthrough knowledge of silica carriers as novel materials, which has great value in giving instructions for the development of other novel nanomaterials and to promote their application for loading and delivering water-insoluble drugs.

## 2. Experimental section

### 2.1 Materials

Tetramethoxysilane (TMOS, ≥99%) and PEI (≥98%) were purchased from Aladdin (Shanghai, China). Polyvidone (PVP) K30 (≥99%) was provided by Anhui Shanhe Pharmaceutical Excipients Co., Ltd. ITZ (≥99%) was purchased from Dalian Meilun Biotechnology Co., Ltd. The cell experimental agents were all purchased from Merck & Co. Life Science Technology (Nantong) Co., Ltd. Other chemical agents, including tween 20, tween 40, tween 80, ethanol, methanol, acetone, monopotassium phosphate, sodium hydroxide, ammonium molybdate, citric acid, ascorbic acid, pyrene, ammonium molybdate, and anhydrous ethanol, were purchased from the Damao chemical reagent factory. Deionized water was prepared by ion exchange. The caco-2 cell line was provided by Shenyang Pharmaceutical University.

### 2.2 Preparation of ITZ micelles

First, 800 mg tween 20, tween 40, and tween 80 were respectively weighed in an eggplant-shaped bottle, and 400 mg ITZ ethanol solution was then added. The mixtures were stirred until completely dissolved. The organic solvent was evaporated using rotary evaporation at 60 °C. The residual solvent was dried under vacuum for 3 h to obtain a transparent drug polymer mixture film. The mixed membrane was preheated in a water bath at 60 °C, and 4 ml of PEI water solution was added, which was hydrated for 10 min. Next, 60 mg PVP K30 was added to obtain the ITZ micelles. After, 0.1 ml prepared micelle solution was diluted by 50 ml methanol. The solution was filtered through a microporous membrane and was measured at 262 nm using a UV spectrophotometer.

### 2.3 Preparation of the drug-loaded silica xerogel

The prepared micelle solution (containing 100 mg of drug) was added into an EP tube, and 0.5 ml TMOS was added. After solidification and drying, the sample was obtained. The carrier solutions that applied tween 20, tween 40, or tween 80 as the assistant agent for forming the silica frame were named as M20-Silica xerogel, M40-Silica xerogel, and M80-Silica xerogel, respectively. In order to figure out how the TMOS amount affected the drug release, a micelle solution (containing 100 mg of the drug) with 0.25 ml TMOS was also mixed to prepare M20′-Silica xerogel, M40′-Silica xerogel, and M80′-Silica xerogel samples, respectively. A certain amount of ITZ-loaded carrier was dissolved in 50 ml methanol under ultrasound, filtered through a 0.45 μm microporous membrane, and the UV absorbance was measured at 262 nm to calculate the drug-loading capacity.

### 2.4 Preparation of the drug-loaded blank silica xerogel

First, 400 mg ITZ was precisely weighed into an EP tube and dissolved in 12 ml anhydrous ethanol. Afterwards, 60 mg PVP K30 was added and the system was shaken well. The above solution was mixed with 4 ml PEI solution, and 0.5 ml TMOS was added to form a blank drug-loaded silica xerogel. The blank silica xerogel was designated as B-Silica xerogel. A certain amount of ITZ-loaded carrier was dissolved in 50 ml methanol under ultrasound, filtered through a 0.45 μm microporous membrane, and the UV absorbance was measured at 262 nm, and the drug-loading capacity was calculated.

### 2.5 Scanning electron microscopy (SEM)

SEM was performed with a SURA 35 field emission scanning electron microscope (ZEISS, Germany) to analyze the surface morphology. The samples were mounted onto metal stubs using double-sided adhesive tape and sputtered with a thin layer of gold under vacuum.

### 2.6 Pore-size distribution

The pore-size distribution of the samples was studied by nitrogen adsorption and desorption measurements using a V-Sorb 2800P system (app-one, China). All the samples were degassed under 50 °C vacuum drying for a sufficient time prior to the analysis to remove any adsorbed water.

### 2.7 Carrier cytotoxicity

A caco-2 cell suspension was cultured at a density of 1.0 × 10^5^ per ml on a 12-well Transwell® culture plate. The culture media consisted of Dulbecco's modified eagle's medium with 1% non-essential amino acids, 1% l-glutamine, 100 U ml^−1^ penicillin, 100 mU ml^−1^ streptomycin, and 10% fetal bovine serum. The culture was stored in a 37 °C and 90% (5% CO_2_) relative humidity environment. The culture solution was changed after inoculation for 24 h to remove the residue and any dead cells. Next, the culture solution was changed several times. After the caco-2 cells formed a tight and complete single-cell layer on the Transwell® culture plate, the cell culture medium was discarded, and HBSS (pH 7.4) solution at 37 °C was added into each hole. Here, the cell surface was washed to remove the attachment, and HBSS solution was added and cultured in an incubator at 37 °C for 30 min.

The cellular toxicities of BM-SX and B-SX were evaluated by MTT assays. Briefly, caco-2 cells were grown for 12 h and were then exposed to various concentrations of samples and further incubated for 48 h. The culture medium with the sample was replaced by 100 μl fresh medium without fetal bovine serum, and then 20 μl sterile MTT solution (5 mg ml^−1^) was added to each well, and incubated for an additional 4 h at 37 °C. The media was removed, and 150 ml DMSO was added to each well to dissolve MTT formazan. The absorbance was measured at 570 nm using a BioRadicroplate reader (Model 500, USA). The cell viability rate was calculated finally.

### 2.8 Fourier transform infrared spectroscopy (FTIR) and differential scanning calorimeter (DSC)

FTIR spectra of the ITZ, carriers (B-Silica xerogel, M20-Silica xerogel, M40-Silica xerogel, M80-Silica xerogel), and the drug-loaded carriers (ITZ-loaded B-Silica xerogel, ITZ-loaded M20-Silica xerogel, ITZ-loaded M40-Silica xerogel, and ITZ-loaded M80-Silica xerogel) were measured using a WQF-530 system (Beifen-Ruili, Beijing).

DSC curves of the ITZ, carriers (B-Silica xerogel, M20-Silica xerogel, M40-Silica xerogel, M80-Silica xerogel), and the drug-loaded carriers (ITZ-loaded B-Silica xerogel, ITZ-loaded M20-Silica xerogel, ITZ-loaded M40-Silica xerogel, and ITZ-loaded M80-Silica xerogel) were analyzed using an HSC-4 system (Hengjiu, Beijing). The samples were heated from 60 °C to 200 °C at a rate of 5 °C min^−1^.

### 2.9 *In vitro* release

The *in vitro* releases of the drug-loaded carriers (containing 10 mg ITZ) and 10 mg ITZ were tested using a stirring paddle method with a dissolution tester. The specific working conditions included: stirring speed of 75 rpm, water bath temperature of 37 ± 0.5 °C, dissolution medium of 900 ml pH 6.8 PBS. During the test, 5 ml sample medium was taken at 5, 10, 15, 20, 30, 40, 60, 90, 120, 150, 180, 210, and 240 min and 5 ml fresh dissolution medium was added to retain a consistent volume. The withdrawn medium was filtered through a 0.45 μm microporous membrane and then measured at 219 nm to calculate the cumulative drug-release amount at the different time points.

### 2.10 Carrier regulation

Optimized experimental design (Design Expert, Version 8.0.6, Stat-Ease Inc., Minneapolis, MN) was used to study how the particle diameter and pore diameter affect the drug release (burst release and total release), which could provide further valuable instruction for designing M-Silica xerogels.

### 2.11 Analysis of the M40-Silica xerogel in the dissolution medium

To reveal the existing situation of the M40-Silica xerogel in the dissolution medium, the silica degradation and micelles concentration in the M40-Silica xerogel were both analyzed. For silica degradation, 10 mg M40-Silica xerogel was precisely weighed into a 10 ml EP tube (each time a new tube) and the time was counted from when 5 ml pH 6.8 PBS was added under a stable temperature horizontal shaking bath (37 °C, 100 rpm). The samples were taken out at 5, 10, 15, 20, and 30 min successively and filtered through a 0.45 μm microporous membrane to get 3 ml medium in the EP tube. In the obtained medium, 150 μl ammonium molybdate solution was added and the system was allowed to stand for 10 min. After 10 min, 150 μl mixed acid solution was mixed into the above system and allowed to stand for 25 min. The absorbance of the sample medium was measured using a UV spectrophotometer at the wavelength of 811 nm.

The concentration of micelles in the M40-Silica xerogel was tested using pyrene solution. Here, 50 mg pyrene was weighed and put in a 100 ml volumetric flask. Acetone was added to completely dissolve pyrene to obtain the standard pyrene acetone solution. Next, 1.0 ml pyrene acetone solution was added to a 10 ml sample bottle and then the solvent was completely dried. Afterwards, the sample solutions that were taken from the dissolution medium at 5, 10, 15, 20, and 30 min were respectively added into the dried sample bottle of pyrene. The fluorescence intensity was measured with a microplate reader with the excitation wavelength at 330 nm and the emission wavelength at 390 nm.

## 3. Results and discussion

### 3.1 FTIR and DSC

**Fig. 1:**
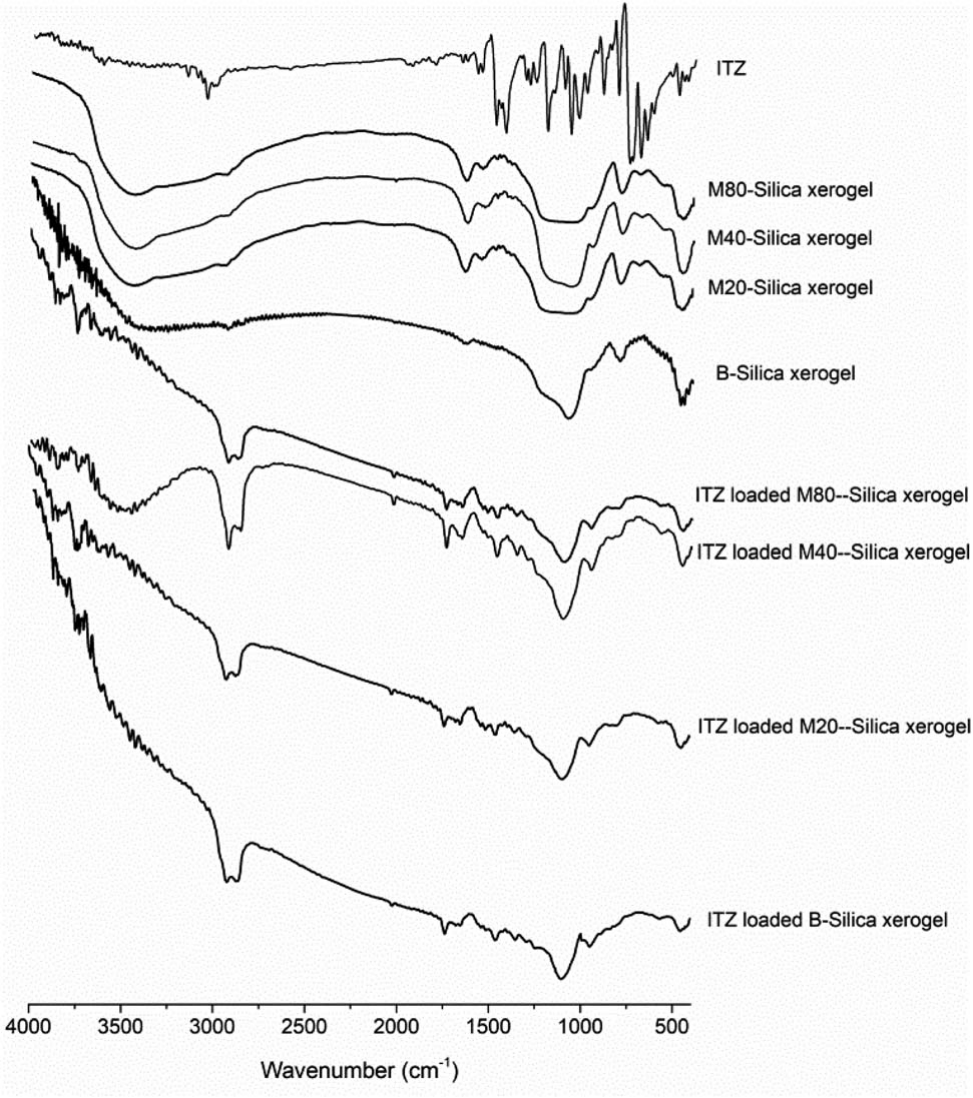
FTIR spectra of the ITZ, B-Silica xerogel, M20-Silica xerogel, M40-Silica xerogel, M80-Silica xerogel, ITZ-loaded B-Silica xerogel, ITZ-loaded M20-Silica xerogel, ITZ-loaded M40-Silica xerogel, and ITZ-loaded M80-Silica xerogel.

According to FTIR results (see Fig. 1), the characteristic peaks of the carriers showed the bending vibration peak of Si–O–Si (462.4 cm^−1^ for B-Silica xerogel, 452.5 cm^−1^ for M20-Silica xerogel, 472.2 cm^−1^ for M40-Silica xerogel, 462.4 cm^−1^ for M80-Silica xerogel), the symmetrical stretching vibration peak of Si–O–Si (787.4 cm^−1^ for B-Silica xerogel, 787.4 cm^−1^ for M20-Silica xerogel, 797.3 cm^−1^ for M40-Silica xerogel, 783.3 cm^−1^ for M80-Silica xerogel), and the asymmetric stretching vibration peak of Si–O–Si (1067.3 cm^−1^ for B-Silica xerogel, 1032.0 cm^−1^ for M20-Silica xerogel, 1072.2 cm^−1^ for M40-Silica xerogel, 1052.5 cm^−1^ for M80-Silica xerogel). Compared to the B-Silica xerogel, the M-Silica xerogel presented extra characteristic peaks of hydrogen bond vibration peaks in the range of 3451–3473 cm^−1^ and carbonyl groups in the range of 1627–1647 cm^−1^ owing to the applied micelles in the carrier. ITZ showed characteristic peaks at 3015.0 cm^−1^ (–CH of benzene ring), 1488.4 and 1438.3 cm^−1^ (C=N stretching), and 1203.6 cm^−1^ (C–N stretching). After loading into the carriers, it was obvious that most of the peaks of ITZ could not be observed, except the C=N stretching of ITZ, which displayed a red-shift, and the –CH of the benzene ring of ITZ, which showed a blue-shift. The presented shifted peaks of ITZ demonstrated that hydrogen bonds had been formed^32^ between ITZ and the carriers.

**Fig. 2:**
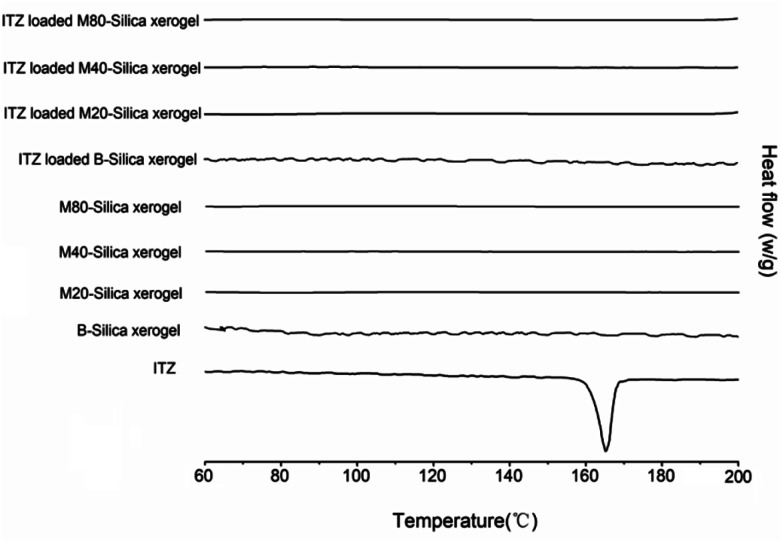
DSC curves of the ITZ, B-Silica xerogel, M20-Silica xerogel, M40-Silica xerogel, M80-Silica xerogel, ITZ-loaded B-Silica xerogel, ITZ-loaded M20-Silica xerogel, ITZ-loaded M40-Silica xerogel, and ITZ-loaded M80-Silica xerogel.

DSC curves were obtained and are shown in Fig. 2. It was obvious that an endothermic peak of ITZ could be observed at 166.2 °C, which belonged to the melting point temperature of ITZ. No peaks could be seen for all the carriers, indicating that the B-Silica xerogel and M-Silica xerogel were amorphous state materials. After loading ITZ into these carriers, no endothermic peak was presented, which confirmed that both the B-Silica xerogel and M-Silica xerogel could convert the drug-crystal state to an amorphous phase.^33^

### 3.2 Materials property of the carriers

**Fig. 3:**
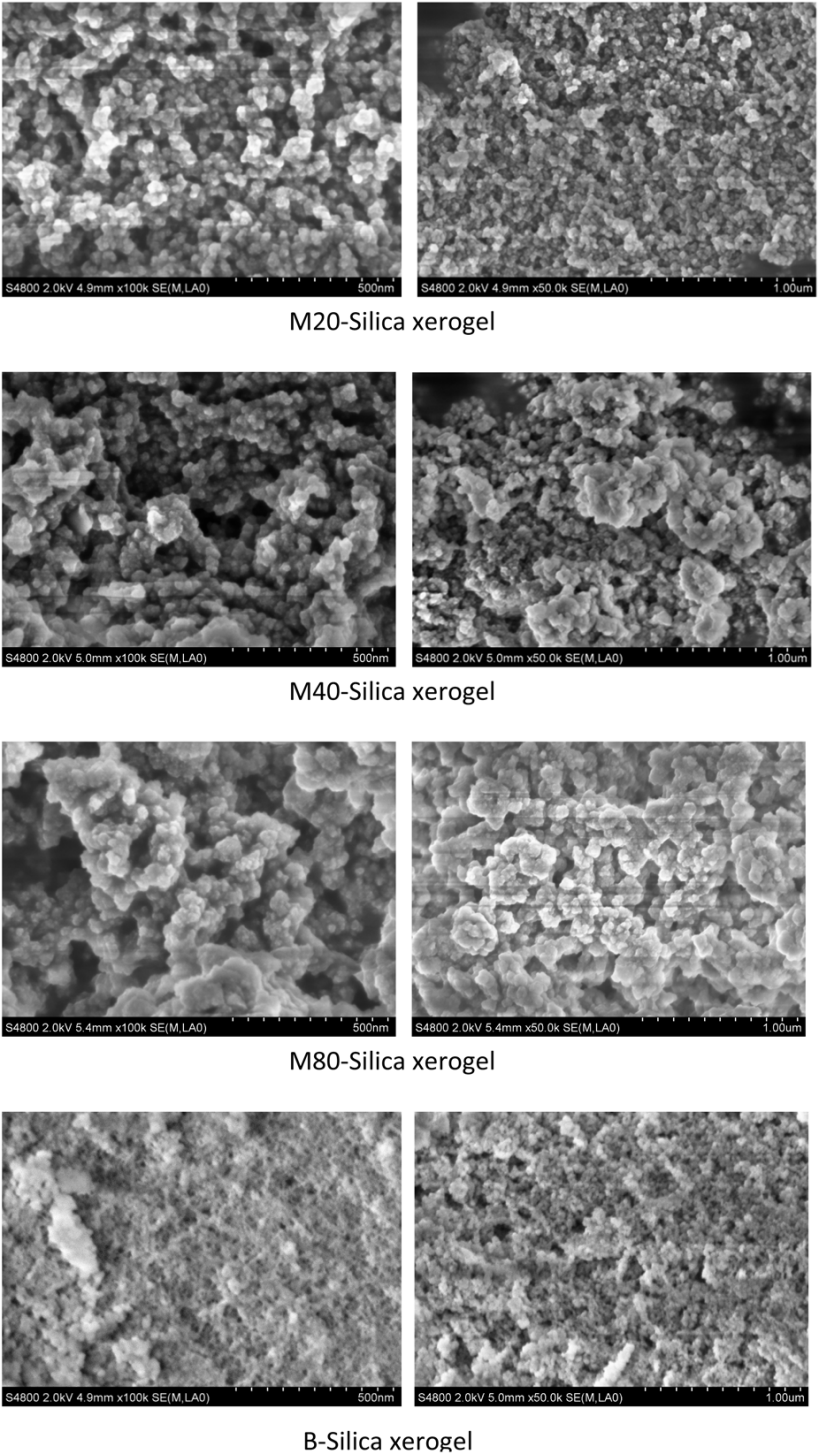
SEM images of the M20-Silica xerogel, M40-Silica xerogel, M80-Silica xerogel, and B-Silica xerogel.

As for the M-Silica xerogel, a one-pot loading method was applied because drug molecules can be incorporated within the carrier during the formation of the silica frame, resulting in its uniform storage in the system. As can be seen from Fig. 3, the particle size of the sub-particles from the M40-Silica xerogel was larger than for the M20-Silica xerogel, and the M80-Silica xerogel was the largest among these three samples, demonstrating that the emulsifying ability directly impacted the particle size of the M-Silica xerogel. The M80-Silica xerogel contained micelles that were formed of tween 80, and there were a large number of micelles since the CMC of tween 80 was low, leading to its large sub-particles in the xerogel. As for the B-Silica xerogel, the silica frame was stacked tightly and the sub-particles were much smaller than for the M-Silica xerogel, demonstrating that the micelles in the synthesized system exerted a crucial function in directing the size of the sub-particles.

**Fig. 4:**
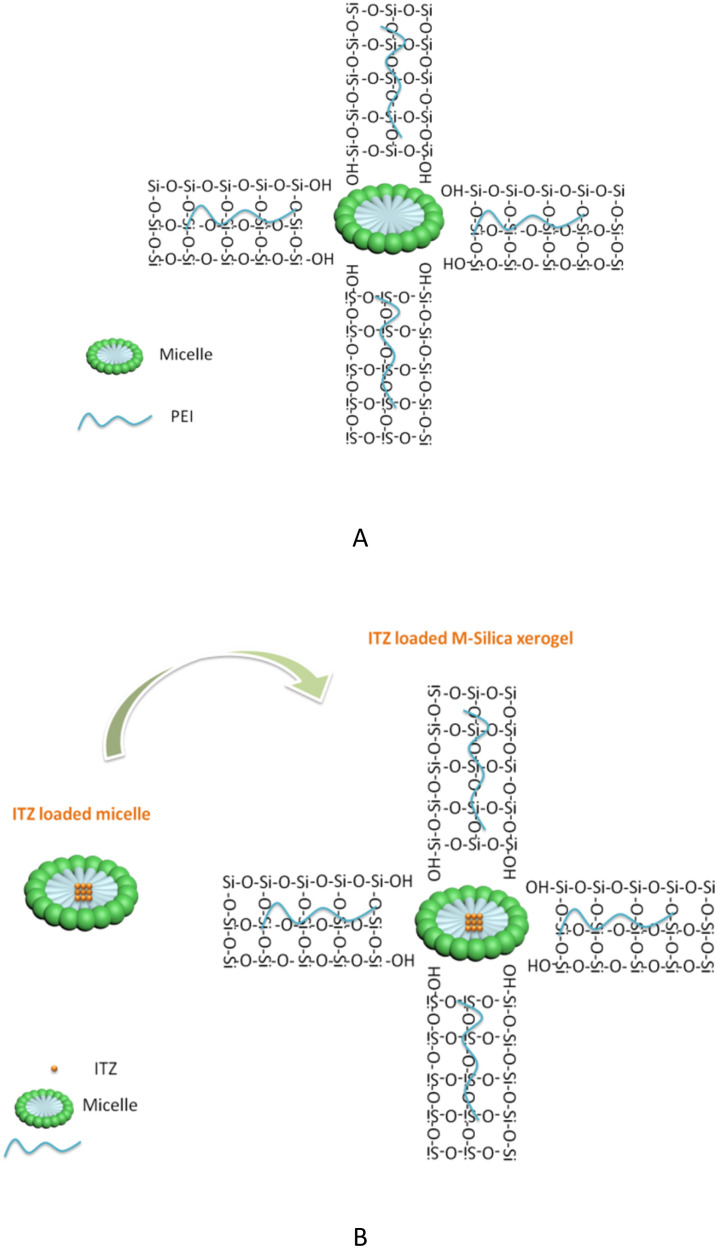
(A) Chemical reaction schematic illustration of the M-Silica xerogel; (B) schematic illustration of the ITZ-loaded M-Silica xerogel.

The M-Silica xerogel was different from the silica particles based on the following aspects: (1) the state of the silica xerogel belonged to a gel, while the silica particles presented as a powder state, caused by their different formation mechanisms. As seen in Fig. 4A, the M-Silica xerogel was formed with the assistance of a surfactant and one polymer PEI (the surfactant formed a micelle as a porous template, then silicon hydroxyl groups interacted with hydrophilic parts of the micelle, and PEI catalyzed the silica polycondensation owing to its amino groups), and the system was aggregated far more tightly due to its high viscosity induced by PEI. However, common silica particles just formed with the template of the surfactant, and the particles accomplished their formation once the interaction between the surfactant and silica source stopped; (2) the porous structure of the silica particles could be normally observed from the SEM, but not for the M-Silica xerogel, which could be ascribed to the gel-formation process of the M-Silica xerogel; (3) a drug could be loaded into the M-Silica xerogel by forming a drug-loaded micelle as a silica frame template, and here, poorly water-soluble ITZ was loaded in the hydrophobic core of the micelle. A schematic illustrating the ITZ-loading process is provided in Fig. 4B.

**Fig. 5:**
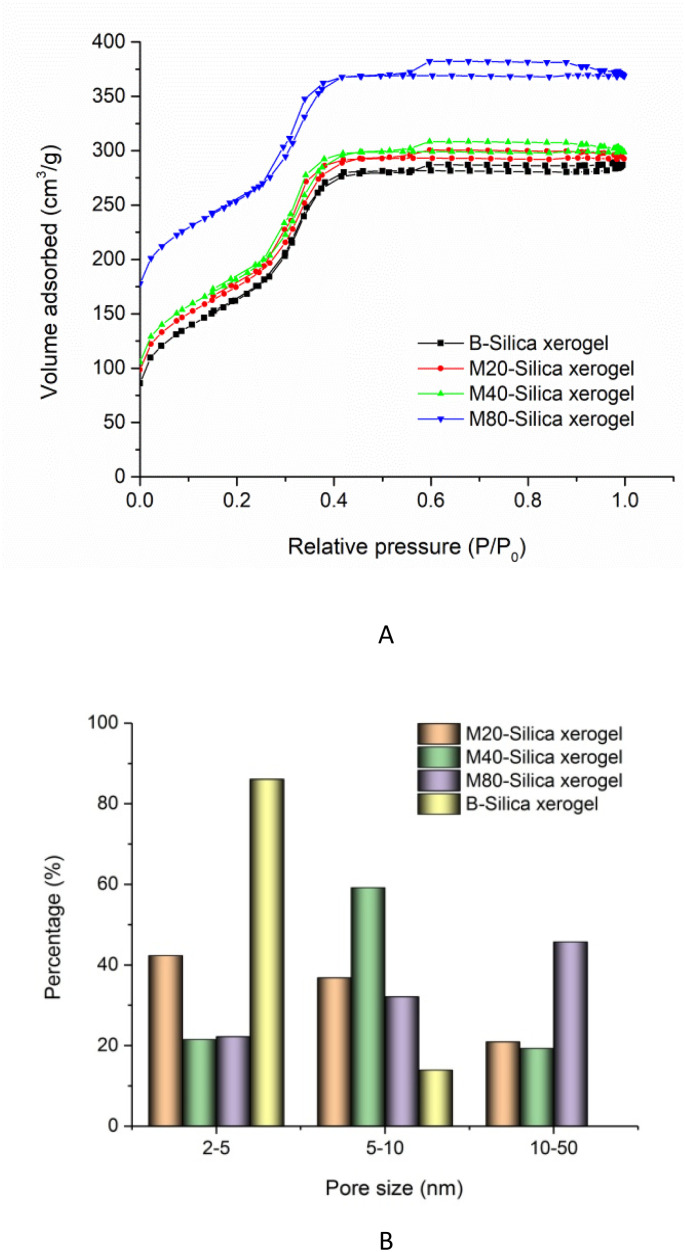
(A) Nitrogen adsorption–desorption isotherms of the M20-Silica xerogel, M40-Silica xerogel, M80-Silica xerogel, and B-Silica xerogel; (B) pore-size conclusion of the M20-Silica xerogel, M40-Silica xerogel, M80-Silica xerogel, and B-Silica xerogel.

In the nitrogen adsorption/desorption isotherm studies of the B-Silica xerogel and M-Silica xerogel (see Fig. 5A), the existence of an hysteresis loop demonstrated their nanopores. Compared to the B-Silica xerogel, there was another hysteresis loop at relative pressures of 0.2 to 0.4, reflecting that there were extra kinds of nanopores present. The specific surface areas of the M20-Silica xerogel, M40-Silica xerogel, M80-Silica xerogel, and B-Silica xerogel were 311.46, 367.21, 437.54, and 302.45 cm^3^ g^−1^, indicating that the addition of tween could affect the specific surface area. Among the M-Silica xerogels, the M80-Silica xerogel had the largest specific surface area. As seen in the pore-size conclusion image of Fig. 5B, the B-Silica xerogel with the smallest sub-particles presented a large number of pores in the range of 2–5 nm and a small number of pores within the 5–10 nm range, showing that the small sub-particles led to a small pore size and low specific surface area, because more of the inter-space was occupied by small sub-particles. On the contrary, the M-Silica xerogel had a large pore size in the range of 10–50 nm, illustrating that the micelles in the system contributed to the large pore size and therefore obtaining a larger specific surface area. The pore-size differences of the three M-Silica xerogels were obvious, whereby small mesopores (2–5 nm) dominated the pore size of the M20-Silica xerogel, medium mesopores (5–10 nm) occupied most of the pore distribution of the M40-Silica xerogel and large mesopores (10–50 nm) shouldered most of the pore distribution for the M80-Silica xerogel. These results demonstrated that tween with a strong emulsifying ability could assist the formation of large mesopores of the silica xerogel since a larger number of micelles led to bigger sub-particles and therefore the space between these sub-particles was larger than for the silica xerogel templated by tween with a weak emulsifying ability.

### 3.3 *In vitro* drug release

**Fig. 6:**
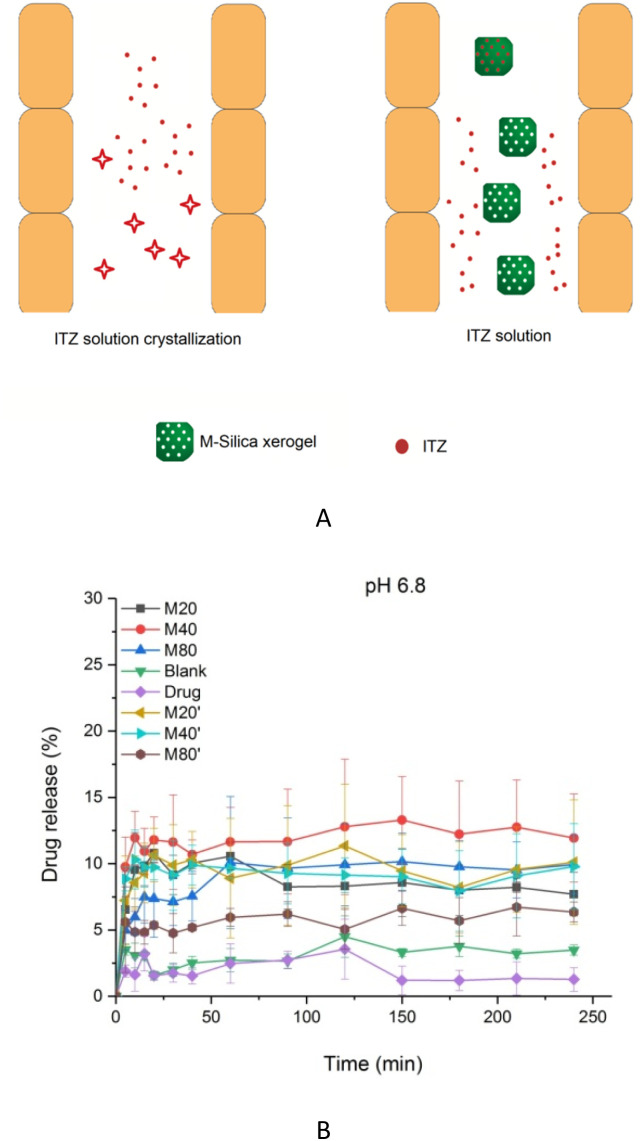
(A) Schematic illustration of the ITZ state in the intestinal tract for ITZ solution and ITZ-loaded M-Silica xerogel solution; (B) *in vitro* drug release of the ITZ-loaded M20-Silica xerogel (M20), ITZ-loaded M40-Silica xerogel (M40), ITZ-loaded M80-Silica xerogel (M80), ITZ-loaded B-Silica xerogel (Blank), ITZ-loaded M20′-Silica xerogel (M20′), ITZ-loaded M40′-Silica xerogel (M40′), ITZ-loaded M80′-Silica xerogel (M80′), and ITZ (Drug) in pH 6.8 medium.

The drug-loading capacities of ITZ in the B-Silica xerogel, M20-Silica xerogel, M40-Silica xerogel, and M80-Silica xerogel were 9.5%, 12.8%, 13.0%, and 12.4%, respectively, possibly demonstrating that the M-Silica xerogel had a better drug-loading ability compared to the B-Silica xerogel, because the micelles could initially entrap more ITZ. Drug release can reflect how the pores of the carrier control the drug-release behavior. ITZ had poor solubility in pH 6.8 medium, resulting in the occurrence of a large amount of crystal ITZ in the small intestine, hindering drug oral absorption. Therefore, maintaining high drug solubility in the gastrointestinal tract turned out to be crucial for achieving superior drug absorption. As can be seen in Fig. 6B, for a low usage of silica xerogel (M20′-Silica xerogel, M40′-Silica xerogel, and M80′-Silica xerogel), the drug-release principle for these three samples was the same, confirming that the mesopores had a great effect on the drug-release behavior. Moreover, all these low assumptions of silica xerogel carriers released less drug molecules than the M20-Silica xerogel, M40-Silica xerogel, and M80-Silica xerogel, demonstrating that a low assumption of the carrier was not able to realize a high drug release, because most of the drug is adsorbed onto the silica frame, which increasingly makes drug release difficult. At pH 6.8, the released ITZ was the lowest among all these samples, which was in accordance with its low solubility in pH 6.8 medium. It should be noted that the low dissolution of ITZ was still sufficient for therapy, because the dosage of ITZ was high (200 mg each time or higher per day) when used in application. To reduce the drug dosage, the ITZ-loaded B-Silica xerogel and ITZ-loaded M-Silica xerogel could achieve a higher drug cumulative release since the carriers were able to enhance the drug solubility in pH 6.8 by dispersing the drug molecules in the mesopores to restrict drug crystallization,^34^ which was in agreement with the DSC result; a schematic illustration of this is displayed in Fig. 6A. To elucidate the drug-release mechanisms, dissolution models were studied, and the drug-release model equations are listed as follows.*R* = *kt* + *C* (Zero-order)ln(1 − *R*) = *kt* + *C* (First-order)*R* = *kt*^1/2^ + *C* (Higuchi)ln *R* = *k* ln *t* + *C* (Ritger–Peppas)where *R* is the fractional release of drug in time *t*, *k* is the rate constant, and *C* is a constant. According to the drug-release model results in Tables 1 and 2, the ITZ-loaded M20-Silica xerogel, ITZ-loaded M20′-Silica xerogel, ITZ-loaded M40′-Silica xerogel, ITZ-loaded M80′-Silica xerogel, and ITZ-loaded B-Silica xerogel fitted the Higuchi model, demonstrating that these drug releases belonged to a diffusion mechanism. The dissolution curves of the ITZ-loaded M40-Silica xerogel, ITZ-loaded M80-Silica xerogel, and ITZ could be fitted to the Ritger–Peppas model, and their *k* values were smaller than 0.45, reflecting that they belonged to a Fickian diffusion mechanism.^35^ In summary, the ITZ-loaded M40-Silica xerogel had the significantly highest drug dissolution in the small intestine and turned out to be the best one among all the xerogels tested here to treat candida infection because its oral absorption was the highest, because most dissolved ITZ could remain in the small intestine. Compared to the B-Silica xerogel, the M-Silica xerogel presented superior drug dissolution owing to the advantages of the micelles in improving the drug solubility.

**Table 1:** Drug-release models results (Zero-order and First-order) of the ITZ-loaded M20-Silica xerogel, ITZ-loaded M40-Silica xerogel, ITZ-loaded M80-Silica xerogel, ITZ-loaded B-Silica xerogel, ITZ, ITZ-loaded M20′-Silica xerogel, ITZ-loaded M40′-Silica xerogel, and ITZ-loaded M80′-Silica xerogel^a^

Sample	Zero-order	First-order
ITZ-loaded M20-Silica xerogel	*R* = 0.0021*t* + 8.079	ln (1 − *R*) = −0.00002*t* − 0.085
	*r* ^2^ = 0.0043	*r* ^2^ = 0.0030
ITZ-loaded M40-Silica xerogel	*R* = 0.0178*t* + 9.442	ln(1 − *R*) = −0.0002*t* − 0.100
	*r* ^2^ = 0.2016	*r* ^2^ = 0.2092
ITZ-loaded M80-Silica xerogel	*R* = 0.0231*t* + 5.897	ln(1 − *R*) = −0.0002*t* − 0.061
	*r* ^2^ = 0.4565	*r* ^2^ = 0.4674
ITZ-loaded B-silica xerogel	*R* = 0.0071*t* + 2.228	ln(1 − *R*) = −0.00007*t* − 0.023
	*r* ^2^ = 0.2831	*r* ^2^ = 0.2840
ITZ	*R* = −0.0011*t* + 1.904	ln(1 − *R*) = 0.00001*t* − 0.019
	*r* ^2^ = 0.0095	*r* ^2^ = 0.0097
ITZ-loaded M20′-Silica xerogel	*R* = 0.0112*t* + 7.884	ln(1 − *R*) = −0.0001*t* − 0.083
	*r* ^2^ = 0.1124	*r* ^2^ = 0.1126
ITZ-loaded M40′-Silica xerogel	*R* = 0.0063*t* + 8.158	ln(1 − *R*) = −0.00006*t* − 0.086
	*r* ^2^ = 0.0405	*r* ^2^ = 0.0387
ITZ-loaded M80′-Silica xerogel	*R* = 0.011*t* + 4.311	ln(1 − *R*) = −0.0001*t* − 0.044
	*r* ^2^ = 0.3022	*r* ^2^ = 0.3087

a *r* ^2^ stands for the fitting degree of the equations.

**Table 2:** Drug-release models results (Higuchi and Ritger–Peppas) of the ITZ-loaded M20-Silica xerogel, ITZ-loaded M40-Silica xerogel, ITZ-loaded M80-Silica xerogel, ITZ-loaded B-Silica xerogel, ITZ, ITZ-loaded M20′-Silica xerogel, ITZ-loaded M40′-Silica xerogel, and ITZ-loaded M80′-Silica xerogel^a^

Sample	Higuchi	Ritger–Peppas
ITZ-loaded M20-Silica xerogel	*R* = 0.0014*t*^1/2^ + 0.072	ln *R* = −0.0176 ln *t* − 2.360
	*r* ^2^ = 0.0671	*r* ^2^ = 0.0243
ITZ-loaded M40-Silica xerogel	*R* = 0.004*t*^1/2^ + 0.078	ln *R* = 0.0477 ln *t* − 2.330
	*r* ^2^ = 0.3710	*r* ^2^ = 0.5423
ITZ-loaded M80-Silica xerogel	*R* = 0.0047*t*^1/2^ + 0.041	ln *R* = 0.1698 ln *t* − 3.169
	*r* ^2^ = 0.6807	*r* ^2^ = 0.8629
ITZ-loaded B-Silica xerogel	*R* = 0.0014*t*^1/2^ + 0.018	ln *R* = 0.074 ln *t* − 3.814
	*r* ^2^ = 0.3741	*r* ^2^ = 0.1150
ITZ	*R* = 0.0001*t*^1/2^ + 0.017	ln *R* = −0.0797 ln *t* − 3.690
	*r* ^2^ = 0.0046	*r* ^2^ = 0.0727
ITZ-loaded M20′-Silica xerogel	*R* = 0.0029*t*^1/2^ + 0.066	ln *R* = 0.0398 ln *t* − 2.517
	*r* ^2^ = 0.2634	*r* ^2^ = 0.1793
ITZ-loaded M40′-Silica xerogel	*R* = 0.0019*t*^1/2^ + 0.072	ln *R* = −0.0183*t* − 2.293
	*r* ^2^ = 0.1331	*r* ^2^ = 0.1232
ITZ-loaded M80′-Silica xerogel	*R* = 0.0023*t*^1/2^ + 0.035	ln *R* = 0.0633*t* − 3.133
	*r* ^2^ = 0.4587	*r* ^2^ = 0.4224

a *r* ^2^ stands for the fitting degree of the equations.

Among all these M-Silica xerogels, the M40-Silica xerogel with the largest amount of medium mesopores showed the highest drug cumulative release compared to the M20-Silica xerogel and M80-Silica xerogel, suggesting that medium mesopores were the best for retaining a high drug solubility in pH 6.8 medium. Small mesopores mostly failed in storing drug molecules and the adsorbed drug on the outside layer was hard to dissolve. In large mesopores, drug molecules could not safely be incorporated in the pores, leading to poor drug dissolution. It should be noted that a low assumption of silica xerogel was not favorable for achieving a high drug solubility in pH 6.8 medium, as evidenced by the lower drug release of the M20′-Silica xerogel, M40′-Silica xerogel, and M80′-Silica xerogel compared to the M20-Silica xerogel, M40-Silica xerogel, and M80-Silica xerogel, which hinted that only enough silica frame can realize a high drug solubility in pH 6.8 medium by successfully storing drug molecules in the mesopores to avoid the existence of crystal drug molecules. According to the carrier design results, it was indicated that the design of mesopores of the M40-Silica xerogel should not be so small or so large, since small mesopores were not good for controlling burst release while large mesopores failed to benefit for obtaining a high total drug release. In this case, the M40-Silica xerogel with a larger amount of medium mesopores could achieve a better drug-release behavior. This valuable principle provides a general instruction for designing M40-Silica xerogel as a carrier of poorly water-soluble drugs.

### 3.4 Carrier safety in cells

**Fig. 7:**
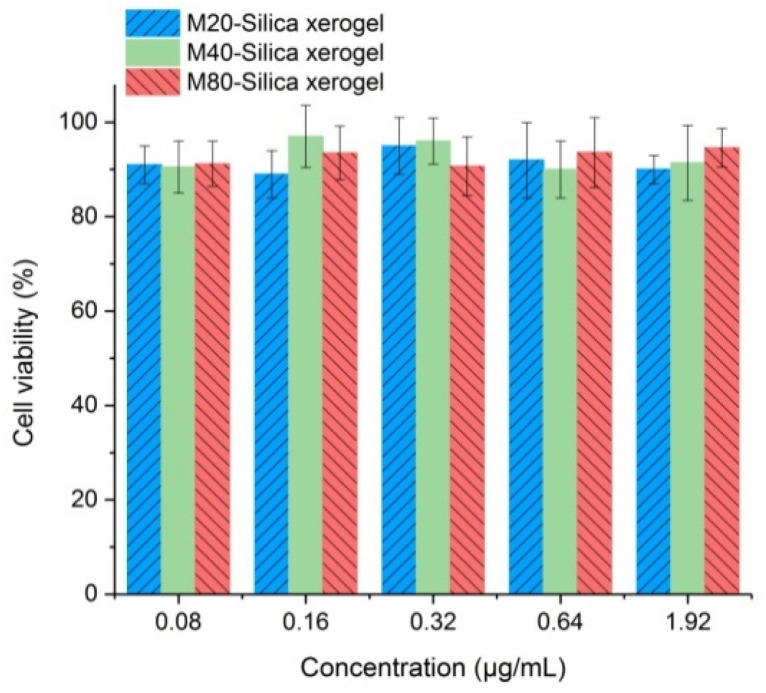
Carrier safety in cells for the M20-Silica xerogel, M40-Silica xerogel, and M80-Silica xerogel.

The application safety in cells is one crucial point to consider for the M-Silica xerogel. Cell viability tests of M20-Silica xerogel, M40-Silica xerogel, and M80-Silica xerogel were conducted with caco-2 cells as model cells (Fig. 7). It turned out that all these samples were safe to be used as drug carriers as evidenced by the fact that the cell viability remained high and no toxicity was observed.

### 3.5 Carrier regulation for releasing ITZ

**Fig. 8:**
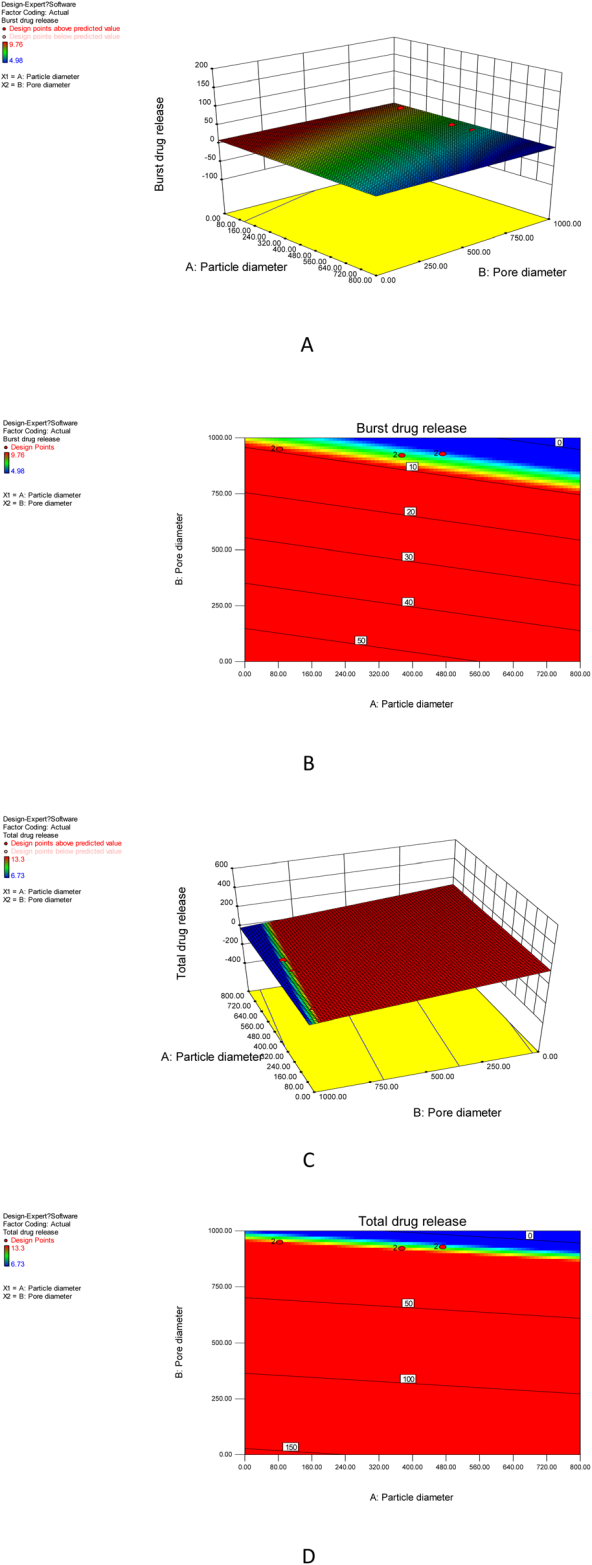
Carrier design results for the M40-Silica xerogel. (A) Response surface plots showing the effects of the independent variables (pore diameter and particle diameter) on the response burst drug release; (B) contour plots showing the effects of the independent variables (pore diameter and particle diameter) on the burst drug release; (C) response surface plots showing the effects of the independent variables (pore diameter and particle diameter) on the total drug release; (D) contour plots showing the effects of the independent variables (pore diameter and particle diameter) on the total drug release.

To figure out how the M40-Silica xerogel property affected ITZ release, carrier design tests were conducted using Design Expert software.^36^ In the carrier design, the particle size and pore size were taken as independent variables, and the burst release together with total release were studied as dependent variables. As can be seen in Fig. 8A and B, the M40-Silica xerogel with a large particle diameter and pore diameter showed a low burst release, demonstrating that the relatively large particle sizes of the M40-Silica xerogel had a superior ability to retain a high drug-storage stability, therefore reducing burst release. As for the total drug-release response in Fig. 8C and D, it was revealed that the M40-Silica xerogel with relatively small particle diameters and pore diameters was favorable for achieving a high total drug release, indicating that micelles with a relatively large particle size and the pore size of the M40-Silica xerogel were not favorable for exerting the function of enhancing drug solubility. This indicated that the micelles were highly dispersed in the carrier and their concentration failed to effectively increase the drug solubility, therefore reducing the total drug release. Based on the above results, it can be concluded that the mesopores of the M40-Silica xerogel had an influence on the drug-release behavior, including on the burst release and total drug release.

### 3.6 Silica degradation in the dissolution medium

The silica degradation of the M40-Silica xerogel was measured and the data were calculated to get its relative score by comparing the data after 5 min with the initial data at 5 min. According to Table 3, silica degraded over time within 40 min, and the micelles amount increased with the degradation of the silica carrier until 30 min. This hinted that the micelles in the M40-Silica xerogel had almost dispersed into the dissolution medium after 30 min, while silica degradation lasted for a much longer time. This result provides a valuable explanation of the dynamic state of the M40-Silica xerogel in the dissolution medium. It was clear that the usage of micelles in the silica xerogel can inhibit drug crystallization during dissolution so as to achieve a superior drug-delivery effect.

**Table 3:** Measurement results of the M40-Silica xerogel degradation in the dissolution medium

Analysis item	Silica degradation (relative score)	Micelle amount (relative score)
5 min	1.0 ± 0.1	1.0 ± 0.2
10 min	3.4 ± 0.2	2.2 ± 0.1
15 min	6.7 ± 0.2	4.5 ± 0.2
20 min	8.9 ± 0.1	6.7 ± 0.1
30 min	11.2 ± 0.2	10.1 ± 0.2
40 min	14.5 ± 0.8	10.1 ± 0.3

## 4. Conclusions

The present work established a new drug carrier with the advantage of micelles and a nanoporous silica xerogel, namely the M-Silica xerogel. Three kinds of M-Silica xerogels, namely M20-Silica xerogel, M40-Silica xerogel, and M80-Silica xerogel, presented different particle sizes and pore-size distributions. It turned out that the particle size of the sub-particles from the M40-Silica xerogel was larger than from the M20-Silica xerogel, and M80-Silica xerogel was the largest among these three samples, hinting that the emulsifying ability directly had an impact on the particle size of the M-Silica xerogel. As for the pore-size distribution, the M-Silica xerogel had a large pore size in the range of 10–50 nm. The M20-Silica xerogel had the largest amount of small mesopores (2–5 nm), while the M40-Silica xerogel had mostly medium mesopores (5–10 nm), and the M80-Silica xerogel presented mostly large mesopores (10–50 nm) distribution. With these significant differences, the M40-Silica xerogel with the largest amount of medium mesopores presented the best ITZ-release behavior, illustrating that medium mesopores facilitated the drug release, possibly because small mesopores impeded drug release and large mesopores were not favorable to retaining an amorphous drug in the pores. The M40-Silica xerogel with superior ITZ release can be outlined as an outstanding carrier and its application should have great value that is worth further investigating.

## Author contributions

Methodology, Xuejun Li.; formal analysis, Dongyan Li.; writing—original draft preparation, Zhining Liu. All authors have read and agreed to the published version of the manuscript.

## Conflicts of interest

There are no conflicts to declare.

## Supplementary Material

## References

[cit1] Jia Z., Lin P., Xiang Y., Wang X., Wang J., Zhang X., Zhang Q. (2011). A novel nanomatrix system consisted of colloidal silica and pH-sensitive polymethylacrylate improves the oral bioavailability of fenofibrate. Eur. J. Pharm. Biopharm..

[cit2] Mellaerts R., Mols R., Jammaer J. A. G., Aerts C. A., Annaert P., Humbeeck J. V., Mooter G. V. d., Augustijns P., Martens J. A. (2008). Increasing the oral bioavailability of the poorly water soluble drug itraconazole with ordered mesoporous silica. Eur. J. Pharm. Biopharm..

[cit3] Mishra J., Adam B., Thomas R., Holger G., Korbinian L. (2019). Whey proteins as stabilizers in amorphous solid dispersions. Eur. J. Pharm. Sci..

[cit4] Karasulu H. Y., Gundogdu E., Turgay T., Turk U. O., Apaydin S., Şimsir I. Y., Yilmaz C., Karasulu E. (2016). Development and Optimization of Self-emulsifying Drug Delivery Systems (SEDDS) for Enhanced Dissolution and Permeability of Rosuvastatin. Curr. Drug Delivery.

[cit5] Chang D., Gao Y., Wang L., Gan L., Chen Y., Wang T., Tao W., Lin M., Huang L., Zeng X. (2016). Polydopamine-based surface modification of mesoporous silica nanoparticles as pH-sensitive drug delivery vehicles for cancer therapy. J. Colloid Interface Sci..

[cit6] Kresge C. T., Leonowicz M. E., Roth W. J., Vartuli J. C., Beckt J. S. (1992). Ordered mesoporous molecular sieves synthesized by a liquid-crystal template mechanism. Nature.

[cit7] Lu T., Li G., Li J. (2020). Biomimetic silica xerogel regulates indometacin release and oral bioavailability by virtue of chiral pores. Microporous Mesoporous Mater..

[cit8] Juere E., Kleitz F. (2018). On the nanopore confinement of therapeutic drugs into mesoporous silica materials and its implications. Microporous Mesoporous Mater..

[cit9] Hei M., Wang J., Wang K., Zhu W., Ma P. X. (2017). Dually responsive mesoporous silica nanoparticles regulated by upper critical solution temperature polymers for intracellular drug delivery. J. Mater. Chem. B.

[cit10] Jiang Q., Wu L., Zheng Y., Xia X., Zhang P., Lu T., Li J. (2021). Biomimetic micellar mesoporous silica xerogel performs superior nitrendipine dissolution, systemic stability and cellular transmembrane transport. Mater. Sci. Eng., C.

[cit11] Zhang R., Hua M., Liu H., Li J. (2021). How to design nanoporous silica nanoparticles in regulating drug delivery:Surface modification and porous control. Mater. Sci. Eng. B.

[cit12] Zhang R., Wang X., Fan N., Li J. (2021). Enhanced anticancer performances of doxorubicin loaded macro-mesoporous silica nanoparticles with host-metal-guest structure. Microporous Mesoporous Mater..

[cit13] Liu R., Wang X., Fan N., Song H., Ma P., Li C., Li J., Sun J. (2021). Superiority of Chiral-handed mesoporous silica nanoparticles in delivering Nimesulide. Mater. Sci. Eng. B.

[cit14] Shakeran Z., Keyhanfar M., Varshosaz J., Sutherland D. S. (2021). Biodegradable nanocarriers based on chitosan-modified mesoporous silica nanoparticles for delivery of methotrexate for application in breast cancer treatment. Mater. Sci. Eng., C.

[cit15] Maria R. J., Jixi Z., Mika L., Cecilia S. (2016). Mesoporous Silica Nanoparticles in Tissue Engineering-a Perspective. Nanomedicine.

[cit16] Liu S.-H., Kuok C.-H. (2018). Preparation of stable tetraethylenepentamine-modified ordered mesoporous silica sorbents by recycling natural Equisetum ramosissimum. Chemosphere.

[cit17] Choonara Bibi F., Choonara Y. E., Kumar P., Divya B., Lisa Cd. T., Viness P. (2014). A review of advanced oral drug delivery technologies facilitating the protection and absorption of protein and peptide molecules. Biotechnol. Adv..

[cit18] Li X., Pan L., Shi J. (2015). Nuclear-Targeting MSNs-Based Drug Delivery System: Global Gene Expression Analysis on the MDR-Overcoming Mechanisms. Adv. Healthcare Mater..

[cit19] Zhang Y. Z., Zhi Z., Jiang T. Y. (2010). Spherical mesoporous silica nanoparticles for loading and release of the poorly water -soluble drug telmisartan. J. Controlled Release.

[cit20] Geng H., Zhao Y., Liu J., Cui Y., Wang Y., Zhao Q., Wang S. (2016). Hollow mesoporous silica as a high drug loading carrier for regulation insoluble drug release. Int. J. Pharm..

[cit21] Gothwal A., Khan I., Gupta U. (2016). Polymeric Micelles: Recent Advancements in the Delivery of Anticancer Drugs. Pharm. Res..

[cit22] Arranja A., Schroder A. P., Schmutz M., Waton G., Schosseler F., Mendes E. (2014). Cytotoxicity and internalization of Pluronic micelles stabilized by core cross-linking. J. Controlled Release.

[cit23] Kirtane Ameya R., Kalscheuer Stephen M., Jayanth P. (2013). Exploiting nanotechnology to overcome tumer drug resistance:Challenges and opportunities. Adv. Drug Delivery Rev..

[cit24] Bodratti Andrew M., Paschalis A. (2018). Amphiphilic block copolymers in drug delivery: advances in formulation structure and performance. Expert Opin. Drug Delivery.

[cit25] Alakhova Daria Y., Zhao Y., Shu L., Kabanov Alexander V. (2013). Effect of doxorubicin/pluronic SP1049C on tumorigenicity,aggressiveness, DNA methylation and stem cell markers in murine leukemia. PLoS One.

[cit26] Yokoyama M. (2014). Polymeric micelles as drug carriers: their lights and shadows. J. Drug Targeting.

[cit27] Cheng X., Yan H., Jia X., Zhang Z. (2016). Preparation and in vivo/in vitro evaluation of formononetin phospholipid/vitamin E TPGS micelles. J. Drug Targeting.

[cit28] Li J., Xu L., Liu H., Wang Y., Wang Q., Chen H., Pan W., Li S. (2014). Biomimetic synthesized nanoporous silica@poly(ethyleneimine)s xerogel as drug carrier: Characteristics and controlled release effect. Int. J. Pharm..

[cit29] Sojakova M., Liptajova D., Borovsky M., Subik J. (2004). Fluconazole and itraconazole susceptibility of vaginal yeast isolates from Slovakia. Mycopathologia.

[cit30] Costa M., Passos X. S., Miranda A. T. B., de Araújo R. S. C., Paula C. R., Silva M. d. R. R. (2004). Correlation of *in vitro* itraconazole and fluconazole susceptibility with clinical outcome for patients with vulvovaginal candidiasis. Mycopathologia.

[cit31] Tsang D., Haddad S., Sternlieb M. (2022). Laryngeal blastomycosis with subsequent heart failure from itraconazole therapy. IDcases.

[cit32] Song B., Wang J., Lu Si-J., Shan Li-Na (2020). Andrographolide solid dispersions formulated by Soluplus to enhance interface wetting, dissolution, and absorption. J. Appl. Polym. Sci..

[cit33] Zhou J., Zhu F., Li J., Wang Y. (2018). Concealed body mesoporous silica nanoparticles for
orally delivering indometacin with chiral recognition function. Mater. Sci. Eng. C.

[cit34] Fan N., Liu R., Ma P., Wang X., Li C., Li J. (2019). The On-Off chiral mesoporous silica nanoparticles for delivering achiral drug in chiral environment. Colloids Surf., B.

[cit35] Serra L., Doménech J., Peppas N. A. (2006). Drug transport mechanisms and release kinetics from molecularly designed poly(acrylic acid-g-ethylene glycol) hydrogels. Biomaterials.

[cit36] Li J., Wang H., Li H., Xu L., Guo Y., Lu F., Pan W., Li S. (2016). Mutual interaction between guest drug molecules and host nanoporous silica xerogel studied using central composite design. Int. J. Pharm..

